# Unraveling the bacterial composition of a coral and bioeroding sponge competing in a marginal coral environment

**DOI:** 10.3389/fmicb.2025.1550446

**Published:** 2025-10-02

**Authors:** Sambhaji Mote, Kalyan De, Mandar Nanajkar, Vishal Gupta

**Affiliations:** ^1^CSIR–National Institute of Oceanography, Dona Paula, India; ^2^Academy of Scientific and Innovative Research (AcSIR), Ghaziabad, India; ^3^Climate Change Cluster, University of Technology Sydney, Ultimo, NSW, Australia

**Keywords:** Nearshore reef, coral eroding sponge, bacterial diversity, amplicon sequencing, Indian Ocean

## Abstract

The newly described bioeroding sponge *Cliona thomasi*, part of the *Cliona viridis* complex, is contributing to coral decline in the central eastern Arabian Sea, the West Coast of India. While its morphological and allelopathic mechanisms in coral invasion are well investigated, the role of its microbial communities in spatial competition is underexplored. This study focuses on the coral *Turbinaria mesenterina* and sponge *C. thomasi,* both known for their distinct symbiotic associations with Symbiodiniaceae. A 16S rRNA V3–V4 amplicon next-generation sequencing approach, followed by processing through the DADA2 algorithm, was used to analyze the bacterial composition. The results showed higher bacterial richness and diversity in coral samples, identifying 30 distinct phyla, compared to 14 in sponge samples. The coral samples were dominated by Proteobacteria, Actinobacteria, Firmicutes, Cyanobacteria, Planctomycetes, Chloroflexi, and Patescibacteria, while Proteobacteria, Cyanobacteria, Planctomycetes, and Actinobacteria were dominant in the sponge. Enrichment analysis revealed higher dominance of Acidobacteria, Actinobacteria, Chloroflexi, Dadabacteria, Firmicutes, Fusobacteriota, and Patescibacteria in the coral samples, while the sponge samples showed enrichment for Cyanobacteria, Planctomycetes, and Bdellovibrionota. Beta-diversity analysis (PERMANOVA and nMDS) showed significant differences, with an average dissimilarity of 81.44% between sponge and coral samples (SIMPER). These differences highlight variations in microbial profiles between sponges and corals, competing in the same vulnerable environment. Exploring the microbiome aspect, therefore, may elucidate physiological and ecological functions of the holobiont while also representing a health status biomarker for corals, supporting their conservation.

## Introduction

The host–microbe interaction is increasingly recognized as a key framework for understanding organismal function, co-evolution, ecosystem roles, and adaptation to climate change (LaJeunesse et al., 2010; Reveillaud et al., 2014; Parfrey et al., 2018). Marine holobionts, such as corals and sponges, harbor extensive, largely unexplored microbial diversity (Sunagawa et al., 2010; Roughgarden et al., 2017). Coral reefs, often described as the “rainforests of the ocean,” rank among the most diverse and productive ecosystems globally (Plaisance et al., 2011; Fisher et al., 2015) but have suffered severe declines from climate change and human impacts (Hughes et al., 2003; Morrison et al., 2019). Rising sea surface temperatures have triggered mass bleaching events worldwide (Hughes et al., 2018; De et al., 2020, 2021), though some corals exhibit thermal tolerance (Williams and Patterson, 2020). Their persistence is further challenged by competitors, such as cyanobacterial mats, macroalgae, and sponges (Bell et al., 2013; Horwitz et al., 2017; Cruz et al., 2018).

Sponges are important reef inhabitants. Some of the clionaid species have been identified as aggressive space competitors that significantly contribute to reef degradation (Holmes et al., 2009; Fang et al., 2014; Halperin et al., 2016; Schönberg et al., 2017). Both corals and sponges form strong holobionts with diverse microbial assemblages that support host adaptation to extreme environments (Bourne et al., 2016; Sacristán-Soriano et al., 2020; Reigel et al., 2024).

Like coral microbiomes (Wilson et al., 2012; van Oppen and Blackall, 2019; Williams et al., 2024), sponges also host abundant and functionally important microbes, sometimes comprising up to 40% of sponge volume (Webster and Taylor, 2012; Thomas et al., 2016) with ecological and biomedical significance (Pita et al., 2018; Zhang et al., 2022; Williams et al., 2024). Both corals and sponges are considered ecosystem engineers (Bourne et al., 2016; Pita et al., 2018; Camp et al., 2020), and studying their microbial dynamics in shared reef habitats can reveal shifts in diversity and host-associated benefits, and ecological function (Ramsby et al., 2018b; Camp et al., 2020; Sacristán-Soriano et al., 2020).

This study was conducted on marginal patch reefs in the Eastern Arabian Sea, India, an understudied region experiencing high environmental variability and multiple stressors, such as thermal bleaching, sedimentation, eutrophication, and acidification (De et al., 2017, 2020, 2021, 2022; Thinesh et al., 2017). Such suboptimal conditions have favored stress-tolerant coral assemblages that may act as climate refuges (Hughes et al., 2017; Cruz et al., 2018; Lough et al., 2018). The site is dominated by resilient genera such as *Porites*, *Turbinaria*, *Goniopora*, *Siderastrea*, and *Pseudosiderastrea* (Hussain and Ingole, 2020; Hussain et al., 2024), with *Turbinaria mesenterina* being especially abundant (De et al., 2022). This foliose coral thrives in turbid, low-light, and high-sedimentation environments (Sofonia and Anthony, 2008; Hoadley et al., 2016; Hussain et al., 2016) and has shown resistance to bleaching events, including in 2015, when it remained largely unaffected (De et al., 2020, 2022; Hussain and Ingole, 2020). However, these reefs are also subject to disease, algal overgrowth, and bioeroding sponge encroachment (Manikandan et al., 2016; Hussain and Ingole, 2020; Mote et al., 2021a).

Therefore, this study examines the bacterial diversity of two ecologically important and stress-tolerant coral species, namely *T. mesenterina* and the bioeroding sponge *Cliona thomasi,* from this environment to better understand microbial assemblages in marginal, bleaching-impacted reefs.

## Materials and methods

### Study site

The study was conducted at the shallow water near-shore patch coral reef in the Grande Island archipelago, Goa, along the Central West Coast of India in the Eastern Arabian Sea (15,021′14.2′′N, 73045′57.8″E). Additional data on the site and previous surveys revealing the thermal tolerance of corals and sponges are provided in the supplementary file of Mote et al. (2021a).

### Sample collection

**Figure 1:**
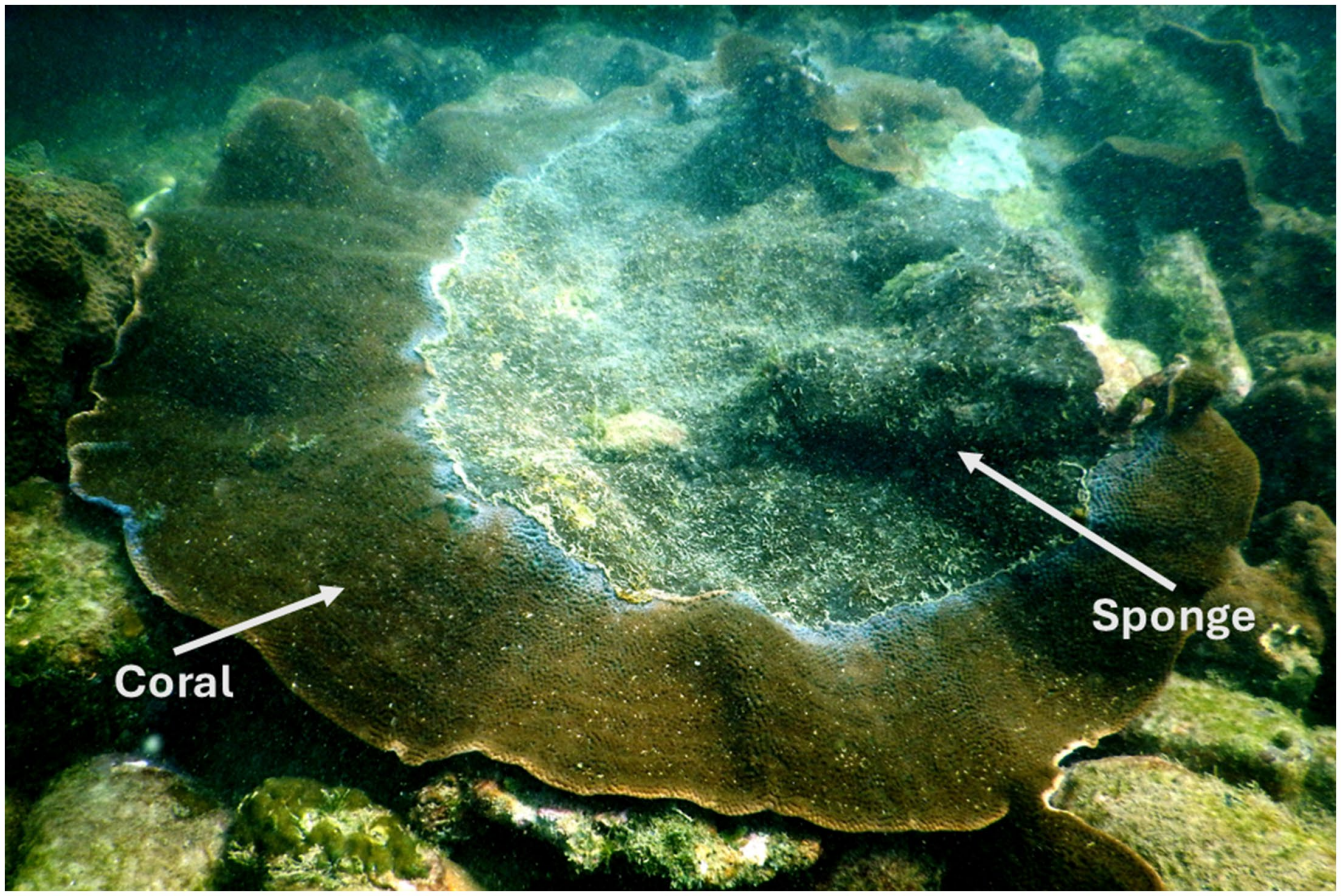
Field photograph of the eroding beta form of *Cliona thomasi* over live coral colonies of *Turbinaria mesenterina* at the study site of Grande Island. Adapted from “Differential Symbiodiniaceae Association With Coral and Coral-Eroding Sponge in a Bleaching Impacted Marginal Coral Reef Environment” by Mote et al. (2021a), licensed under CC-BY 4.0.

This sampling was part of our previously published study (Mote et al., 2021a), and a subset of those samples was utilized in the present study. Coral and sponge samples were collected from a depth of 6–8 m, with each sponge-invaded coral colony separated by a distance of at least 5–10 m. At each sampling point, a small piece of coral and sponge tissue (approximately 10 g) was collected using a preautoclaved hammer and chisel. It was placed individually in a sterile plastic bag. Samples were immediately brought on board, fixed in liquid nitrogen, and transported to the laboratory for further processing. In the laboratory, each sample was stored at −70 °C until DNA extraction. A field photograph of sponge encrustation on coral is shown in Figure 1.

### DNA extraction and high-throughput amplicon sequencing

The collected coral and sponge samples were homogenized in liquid nitrogen and processed for DNA extraction using a tissue DNA extraction kit, as directed by the manufacturer (Invitrogen, CA, USA). The 16S rRNA V3-V4 hypervariable region of the prokaryotic 16S rRNA gene was amplified by PCR for bacterial community analysis (Muyzer et al., 1993; Li et al., 2009). A standard approach was used to create Illumina MiSeq 16S rRNA amplicon libraries (New England Biolabs, Frankfurt, Germany). The libraries were tested for quality using an Agilent 2,100 Bioanalyzer, and the samples were sequenced using 2 × 300 paired-end chemistry (MiSeq Reagent Kit, San Diego, CA, USA).

### Bioinformatics analysis

The sequencing data were processed in R using the DADA2 tool to assemble raw reads and microbial annotation (Bolyen et al., 2019). The primers and adapters were removed before processing the sequence data in Cutadapt (Martin, 2011). After demultiplexing and inspection of the read quality, reads were trimmed with trunclan = c (240, 180) with maxEE = c (2, 2). Reads were then dereplicated, merged, and filtered to remove chimeras and subsequently clustered into amplicon sequence variants (ASVs). The resultant ASVs were assigned to the bacterial taxonomy using a Bayesian classifier with a pretrained Silva-132 database with a minimum bootstrap confidence set to 80. After taxonomic assignments, the ASVs annotated to mitochondria, chloroplast regions, and also Archaea were removed.

### Statistical analysis

The vegan package v.2.5–6 in R (Oksanen et al., 2013) was used to calculate the alpha-diversity indices such as ASV richness, Shannon, Chao1, and Simpson indices, and to prepare a rarefaction curve for the investigated samples. Furthermore, the beta-diversity measures to test the statistical difference of bacterial community composition among the samples were determined by applying permutational multivariate analysis of variance (PERMANOVA) with 999 permutations using the Bray–Curtis dissimilarity matrix in PRIMER v7 (Clarke and Gorley, 2015). The bacterial community composition was ordinated using non-metric multidimensional scaling (nMDS), as implemented in the vegan package in R using the metaMDS function (Oksanen et al., 2013). Differential abundance analysis was carried out using the linear discriminant analysis effect size (LEfSe) analysis. The distinct profiles of core bacterial abundances were selected based on an LDA score of >2 and a *p*-value of < 0.05 from the LEfSe. Predictive functional analysis was carried out using Tax4Fun2 (Wemheuer et al., 2020), producing a Kyoto Encyclopedia of Genes and Genomes (KEGG) Orthology (KO) table that was further analyzed in MicrobiomeAnalyst for pathway annotation (Lu et al., 2023).

## Results

A total of 2.89 million raw reads were generated from 10 samples (Supplementary Table 1). Following quality filtering, 654,529 reads were taxonomically classified, resulting in 7,613 ASVs after clustering and chimera removal (Supplementary Table 2). The ASV distribution, indicating sequencing depth and diversity for coral and sponge samples, is illustrated in the rarefaction curve (Supplementary Figure 1). The rarefaction curve indicated higher sequencing coverage and taxonomic assignments in coral samples compared to sponge samples (Supplementary Figure 1). The identified ASVs richness value ranging from 1,118 to 1,320 for corals and 260–402 for sponges (Supplementary Table 2). The alpha diversity indices indicated greater diversity and richness in coral samples relative to sponge samples (Supplementary Table 2). Shannon’s diversity index for sponge samples ranged from 3.51 to 3.87, while coral samples exhibited a range of 5.11–5.70 (Supplementary Table 2). Simpson’s diversity values were found to be between 0.93 and 0.96 for sponge samples and between 0.98 and 0.99 for coral samples (Supplementary Table 2).

**Figure 2:**
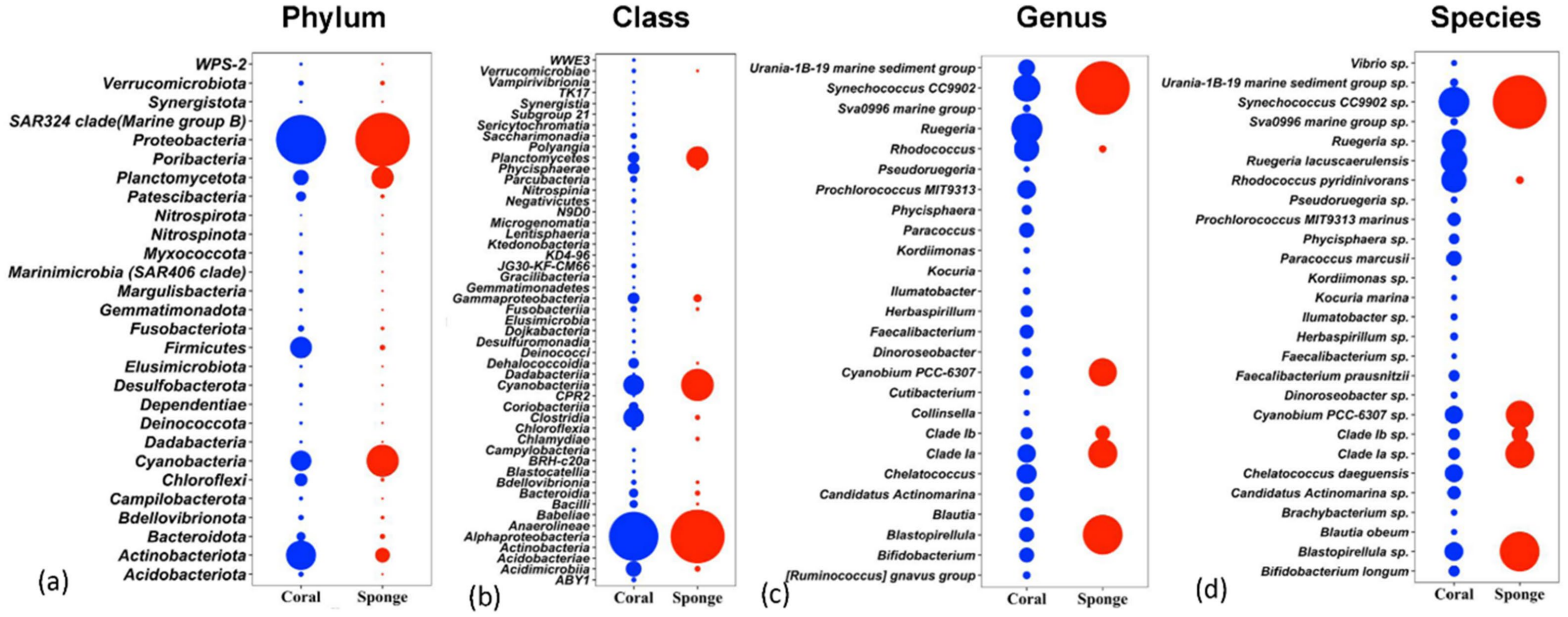
Relative abundances of bacterial communities associated with coral and sponge samples at four taxonomic levels: **(a)** phylum, **(b)** class, **(c)** genus, and **(d)** species. The plots display the top taxa (based on relative abundance) identified across all samples.

**Figure 3:**
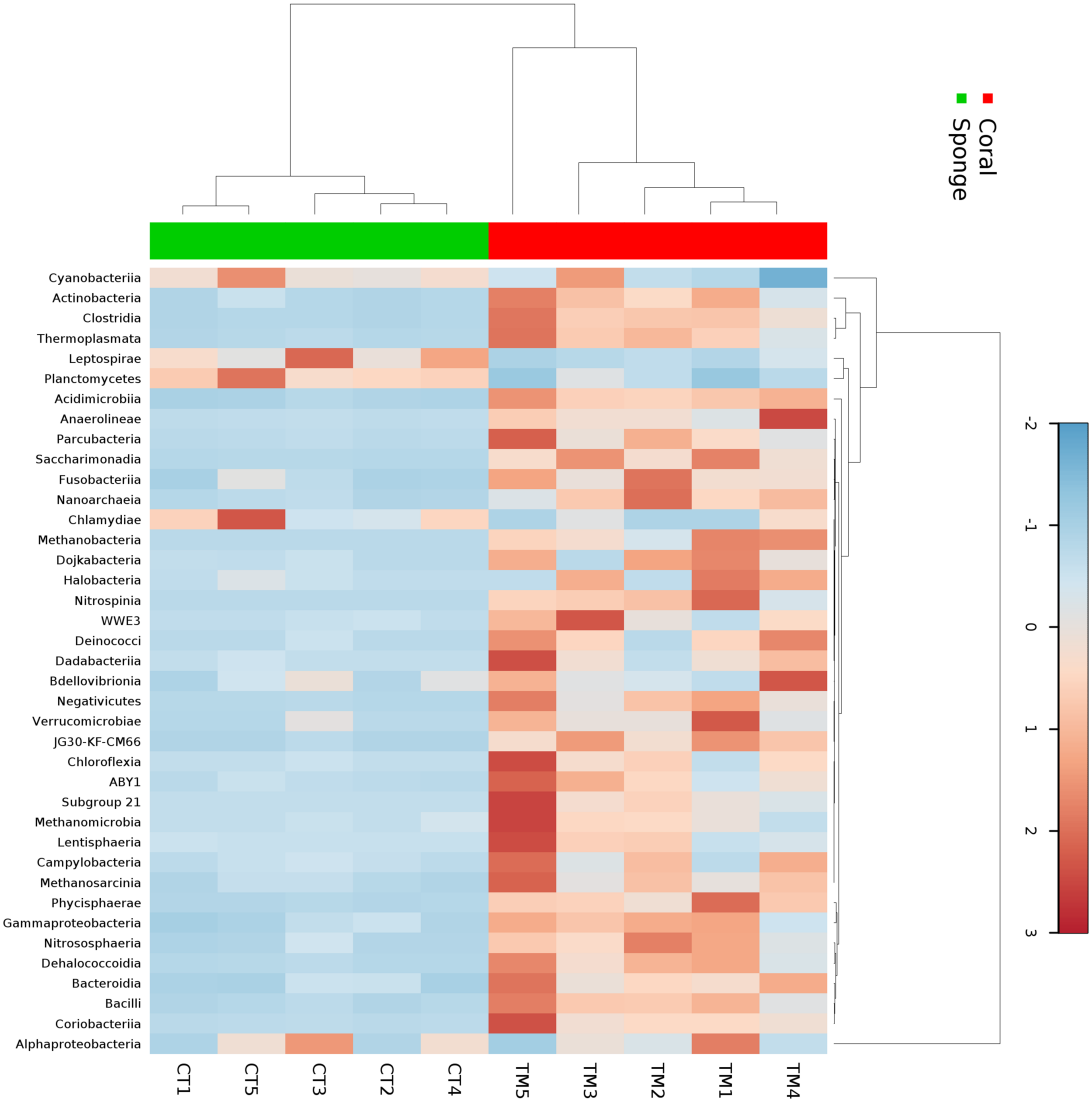
Heatmap showing the taxonomic distribution of bacterial abundance at the class level across five sponge samples and five coral samples. The color gradient represents the relative abundance of each bacterial class, with darker shades indicating higher abundance. Hierarchical clustering was performed using the Bray–Curtis dissimilarity metric to group samples and taxa based on similarity in microbial community composition.

The affiliation of ASVs to bacterial taxa revealed 30 phyla in coral samples vs. 14 in sponge samples (Figure 2a). Dominant bacterial phyla in coral were identified as Proteobacteria (48.99 ± 11.51%), Actinobacteria (16.11 ± 4.64%), Firmicutes (8.06 ± 2.87%), Cyanobacteria (7.22 ± 5.76%), Planctomycetes (3.75 ± 1.75%), Chloroflexi (2.46 ± 0.75%), and Patescibacteria (1.32 ± 0.33%) (Figure 2a). Sponge samples exhibited less diversity with dominant phyla including Proteobacteria (63.44 ± 7.14%), Cyanobacteria (21.44 ± 4.66%), Planctomycetes (9.52 ± 2.32%), and Actinobacteria (3.64 ± 0.75%). Within Proteobacteria, Alphaproteobacteria (51.81 ± 11.71%) and Gammaproteobacteria (2.12 ± 0.52%) were predominant in coral samples (Figure 2b). Conversely, only Alphaproteobacteria (63.19 ± 7.28%) dominated sponge samples (Figure 2b). Following Alphaproteobacteria, coral samples also featured Actinobacteria (12.59 ± 4.62%), Cyanobacteria (7.95 ± 0.63%), Clostridia (7.83 ± 3.16%), Acidimicrobiia (4.20 ± 1.74%), Phycisphaerae (2.19 ± 0.70%), Planctomycetes (1.93 ± 0.38%), Dehalococcoidia (1.57 ± 0.74%), and Coriobacteriia (1.19 ± 0.76%) (Figure 2b). In sponge samples, Cyanobacteria (21.65 ± 4.65%) and Planctomycetes (9.50 ± 2.35%) followed Alphaproteobacteria as the dominant classes. Class-level bacterial diversity is illustrated on a heatmap (Figure 3). The dominant genera in coral samples were Ruegeria (14.97 ± 3.62%), Rhodococcus (13.28 ± 4.18%), Chelatococcus (7.18 ± 3.97%), Paracoccus (4.89 ± 2.64%), Prochlorococcus (4.12 ± 1.83%), Candidatus Actinomarina (3.28 ± 1.06%), and Blastopirellula (2.95 ± 1.34%) (Figure 2c). In contrast, the sponge sample’s dominant genus was Synechococcus (44.33 ± 3.00%), followed by Blastopirellula (23.42 ± 2.36%) (Figure 2c). Species-level changes are depicted in Figure 2d.

**Figure 4:**
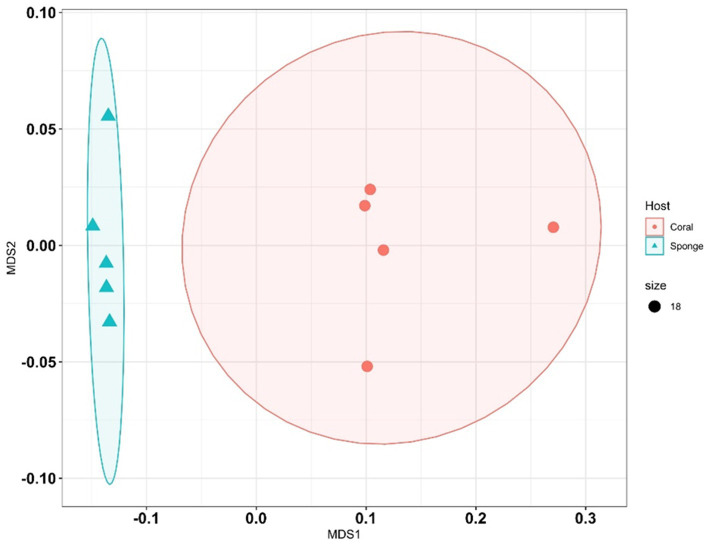
Non-metric multidimensional scaling (NMDS) plots illustrate differences in bacterial community composition between sponge and coral samples. Each point represents a sample, with shapes or colors indicating sample type (sponge vs. coral).

Following ASV annotation, beta-diversity measures were assessed for sponge and coral samples. Beta-diversity analysis via PERMANOVA indicated significant differences between coral and sponge samples (*p* = 0.007, permutation N: 999) (Supplementary Table 3). Multivariate clustering through nMDS corroborated the distinct bacterial community distributions between coral and sponge samples (Figure 4). Further confirmation of bacterial diversity differences was provided by SIMPER analysis, revealing an average dissimilarity of 81.44% between sponge and coral samples (Supplementary Table 4).

**Figure 5:**
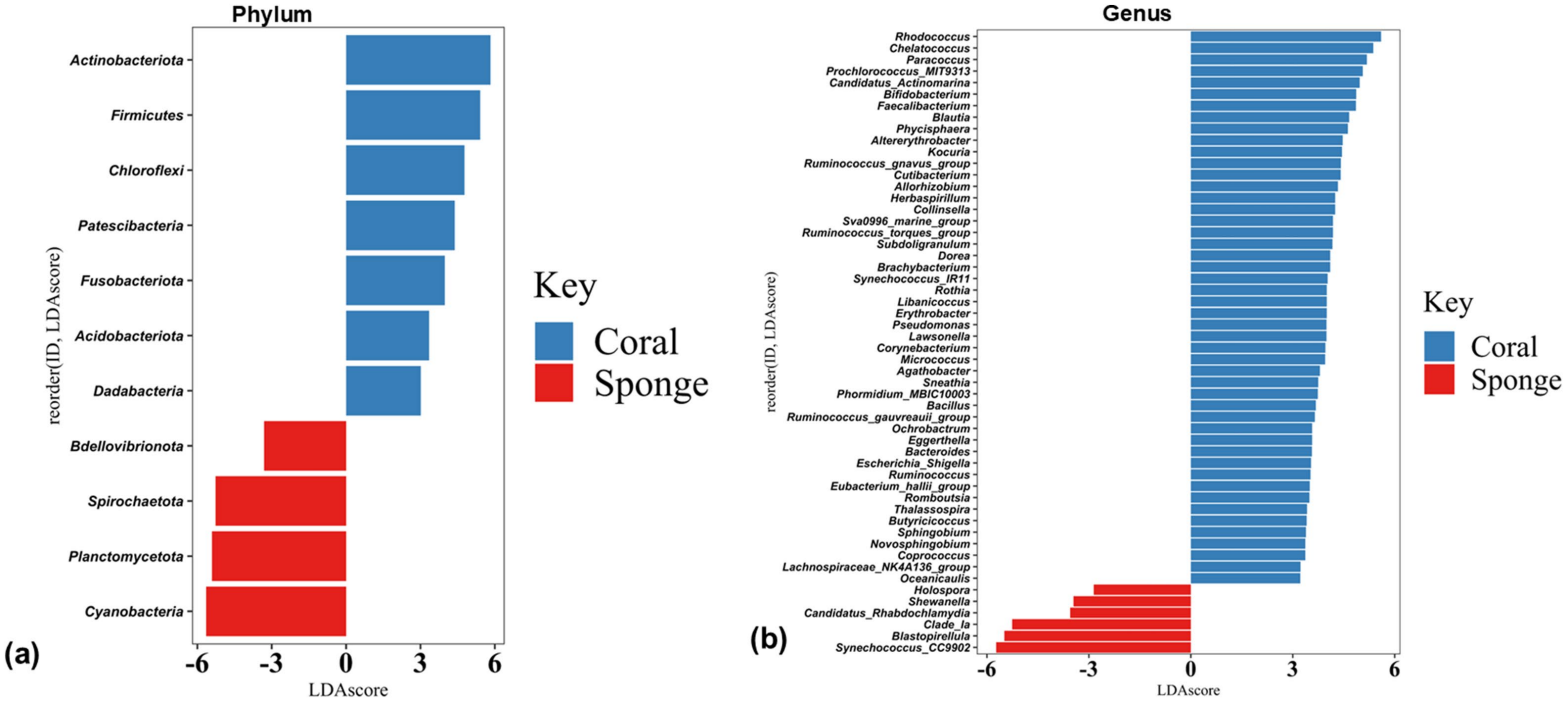
Histogram plots of linear discriminant analysis (LDA) scores showing bacterial taxa differentially abundant between coral and sponge samples at **(a)** phylum and **(b)** genus level. Only taxa with LDA scores > 2 and *p*-value < 0.05 are shown, with positive LDA values representing bacterial groups enriched in coral samples, while negative values correspond to those enriched in sponge samples.

The disparity in the bacterial communities between coral and sponge hosts was validated by LEfSe analysis (*p* < 0.05, LDA score = 2). Coral samples predominantly featured Acidobacteria, Actinobacteria, Chloroflexi, Dadabacteria, Firmicutes, Fusobacteriota, and Patescibacteria (Figure 5a). Sponge samples were mainly characterized by Cyanobacteria, Planctomycetes, and Bdellovibrionota (Figure 5a). Significant differences in bacterial genera between coral and sponge samples are illustrated in Figure 5b. Dominant genera linked to sponge samples include Synechococcus_CC9902, Blastopirellula, Clade_Ia, and Candidatus-Rhabdochlamydia (Figure 5b). In contrast, genera such as Rhodococcus, Chelatococcus, Paracoccus, Prochlorococcus_MIT9313, and Candidatus-Actinomarina were more prevalent in coral samples (Figure 5b). These findings indicate a reduced bacterial community abundance in sponges compared to corals.

**Figure 6:**
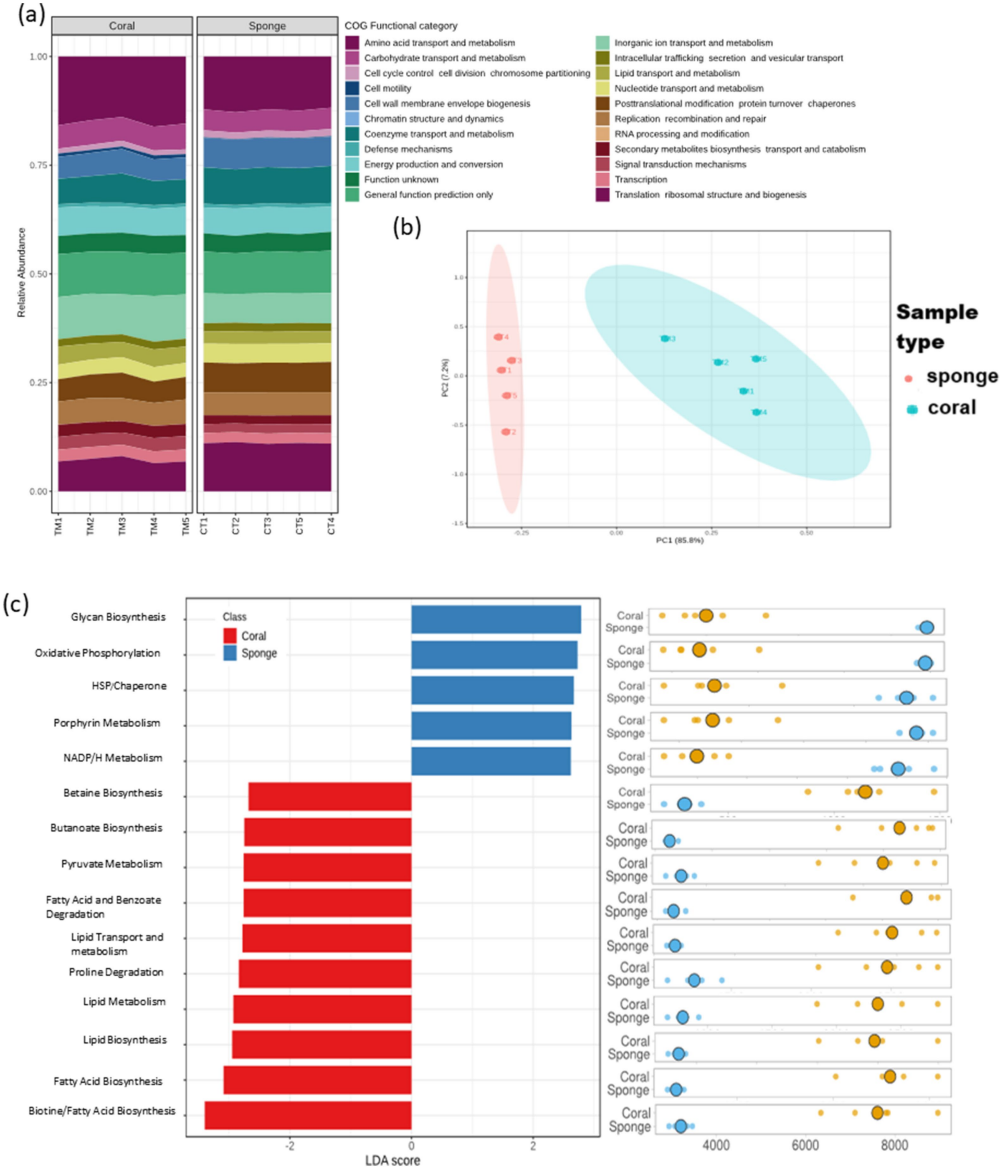
Predictive functional profiling of coral and sponge microbiomes based on amplicon sequencing data. **(a)** Bar chart representing 23 primary functional categories based on Clusters of Orthologous Groups (COG) functional attributes. **(b)** Sample distribution based on KEGG Ortholog (KO) terms using principal component analysis (PCA). **(c)** Differential functional enrichment using LEfSe from major KO terms (LDA score > 2; *p* < 0.05).

The predictive functional analysis identified 3,434 KO terms across the investigated sponge and coral samples. These KO terms were categorized into 23 primary COG functional categories (Figure 6a). The PCA of KO term abundances demonstrated a distribution pattern consistent with ASV analysis (Figure 6b). Differential KO distribution analysis revealed 3,312 KOs varying between the samples. LEfSe analysis identified 202 KOs with significant enrichment (LDA score >2, *p* < 0.05). Sponge samples showed enrichment for 124 KO terms, while coral samples had 78. Despite having fewer enriched KO terms, coral annotations exhibited greater pathway diversity than sponge (Figure 6c). The predominant predictive pathways in sponge samples included glycan biosynthesis, oxidative phosphorylation, porphyrin metabolism, and nicotinamide adenine dinucleotide phosphate (NADPH) metabolism, primarily associated with energy metabolism. Conversely, coral samples displayed a diverse array of enriched predictive pathways encompassing primary and secondary metabolism, such as carbohydrate and lipid metabolism.

## Discussion

In this study, the bacterial communities associated with *T. mesenterina* and *C. thomasi* showed significant differences, despite both organisms inhabiting the same marginal reef environmental conditions. The coral-associated bacteria displayed greater taxonomic richness and diversity, with a broader representation of bacterial phyla and functional pathways. In contrast, the sponge microbiome was less diverse. The lower microbial abundance (LMA) determined in the sponge in this study corroborates well with previous reports defining *Cliona viridis* complex species as having LMA (Jeong et al., 2015; Ramsby et al., 2018b; Easson et al., 2020; Sacristán-Soriano et al., 2020).

### Coral bacterial diversity

The coral species *T. mesenterina*, a widely distributed reef-building coral in the Indo-Pacific region, is known for its stress tolerance and resilience (Veron, 2000; Sofonia and Anthony, 2008). In our analysis, *T. mesenterina* harbored a taxonomically rich community spanning 30 bacterial phyla, with high alpha diversity. Proteobacteria dominated the assemblage (~53% of total abundance), in line with reports from other reef-building and soft corals (Qi et al., 2022; Mohamed et al., 2023). Within this phylum, Alphaproteobacteria were found to be dominant and are consistent with previous findings, where they contribute to growth support, nutrient cycling, and the production of bioactive compounds such as vitamin B₁₂ (Maire et al., 2021; Lin et al., 2022; Shoguchi et al., 2024). Although many Alphaproteobacteria ASVs could not be resolved at the genus level, *Ruegeria* (family Rhodobacteraceae) emerged as the dominant taxon. Members of this genus are recognized for their potential role in coral health, particularly under heat stress (Rosado et al., 2019).

Actinobacteria formed the second most abundant phylum (~17% of relative abundance), dominated by *Rhodococcus*. Several studies have highlighted the critical role of Actinobacteria in supporting coral survival under heat stress (Li et al., 2023; de Breuyn et al., 2025; Osman et al., 2025). Other notable genera included *Aeromicrobium* and *Streptomyces*, which are known to produce antimicrobial and antifungal compounds in corals, making them promising sources of bioactive metabolites (Mahmoud and Kalendar, 2016; Betancur et al., 2017). Firmicutes were the next most abundant phylum detected in the coral samples, a group often reported to increase in corals experiencing elevated temperatures or in contact with turf and macroalgae (Rajasabapathy et al., 2020).

Cyanobacteria represented approximately 7% of the total relative abundance, ranked after Actinobacteria. Although less abundant, this group plays key ecological roles in reef ecosystems, such as nitrogen fixation, calcification, and decalcification (Lesser et al., 2007; Charpy et al., 2012). Other major phyla detected include Planctomycetes, which are found as the most common associates with corals, either in their healthy or diseased stage (Lage and Bondoso, 2014; Kaboré et al., 2020; Rajasabapathy et al., 2020). Since the corals from the investigated habitat had previously experienced multiple stresses, mainly heat stress (Hussain and Ingole, 2020; Arora et al., 2021; Mote et al., 2021a), the observed microbiome composition likely reflects the same.

### Sponge bacterial diversity

Interestingly, the microbial community composition of *C. thomasi* closely resembled that observed in our previous study conducted at another site along the same coastline, located 100 km away from the current study area (Mote et al., 2021b). Furthermore, the determined bacterial community is comparable with the other reports from the *C. viridis* species complex (Jeong et al., 2015; Ramsby et al., 2018b; Easson et al., 2020; Sacristán-Soriano et al., 2020) and supports that this group has an LMA. Like coral, the investigated sponge samples were found to be dominantly associated with Alphaproteobacteria. Previous studies on the *C. viridis* complex species microbiome from different geographical locations, including those from the Pacific region, have also reported Alphaproteobacteria as a primary inhabitant (Jeong et al., 2015; Ramsby et al., 2018b; Easson et al., 2020; Sacristán-Soriano et al., 2020; Mote et al., 2021b). Various physiological processes of the sponge are known to be governed by the activity of Alphaproteobacteria (Hudspith et al., 2021; Sánchez-Suárez et al., 2022).

The Cyanobacteria were the second most abundant phylum in *C. thomasi*. The cyanobacteria had been reported for their photo-protective effects against intermittent high-light exposure to the sponge (Steindler et al., 2002; Pineda et al., 2016) and are also known to produce cytotoxic secondary metabolites (Teruya et al., 2004; Matthews et al., 2020). Planctomycetes were another dominant bacterial group. This phylum is known to be an important component of the sponge microbiome as well as the broader marine microbial community (Fuerst and Sagulenko, 2011; Lage and Bondoso, 2014; Thomas et al., 2016). Notably, it may have a major role in host resource partitioning, as reported in corals (Turnlund et al., 2023).

### Comparative microbiome analysis of corals and bioeroding sponges from marginal reef

Bioeroding sponges represent a growing concern for coral reef ecosystems, as their abundance over live corals has been reported to increase in many reef regions, largely in response to climate change and other cumulative environmental stressors (Bell et al., 2013, 2018; Carballo et al., 2017). Both the coral and sponge samples were dominated by Proteobacteria, with Alphaproteobacteria accounting for the largest proportion of ASVs. Both the coral species *T. mesenterina* and the sponge *C. thomasi* are known for their symbiotic associations. Our previous study highlighted their distinct endosymbiotic dinoflagellate clades *Durusdinium* and *Gerakladium* in *T. mesenterina* and *C. thomasi*, respectively, within the same habitat (Mote et al., 2021a). Notably, the dominance of Alphaproteobacteria alongside Symbiodiniaceae has been reported as a crucial tripartite interaction involving the coral–sponge host, their algal symbionts, and associated bacterial communities (Matthews et al., 2020).

Cyanobacteria were the second most abundant phylum in sponge samples, with a higher abundance compared to coral tissues. Sponge species with photosymbiotic dinoflagellates from the Pacific region are typically dominated by cyanobacteria (Biggerstaff et al., 2015; Pineda et al., 2017; Ramsby et al., 2018a, 2018b). There is strong evidence that the cyanobacterial symbionts in the sponge support an energy trade-off for the sponge host by facilitating photoacclimatization to site-specific turbidity (Biggerstaff et al., 2015). Another study from the Pacific region showed an increase in cyanobacterial abundance with the bioeroding sponge *Cliona orientalis* as an opportunistic proliferation, supporting the host’s energy requirements. The other dominant phylum in the sponge was Planctomycetes, with significantly higher abundance than in coral, whereas Actinobacteria and Firmicutes were relatively enriched in coral tissues. Such compositional differences may reflect variations in surface morphology, microhabitat conditions, and nutrient utilization strategies.

Predicted functional profiles based on KEGG Orthology (KO) annotations mirrored the taxonomic patterns and revealed distinct clustering of coral and sponge samples (Figure 6b). The coral-associated microbial communities showed a greater diversity of predicted pathways, contributing to their metabolic versatility and rapid responsiveness to environmental changes. However, these microbial communities may also facilitate shifts toward opportunistic states under stress. In contrast, sponge-associated microbes exhibited higher predicted abundances of functions related to structural integrity, redox balance, and resilience to environmental fluctuations, potentially underpinning microbial stability in turbid reef environments (Figure 6a). These functional attributes suggest that sponge-associated microbial communities confer greater stability than those of corals at the studied marginal reef site. This interpretation is supported by field observations indicating an increase in sponge cover relative to coral at the investigated site (Mote et al., 2021a).

## Conclusion

This study demonstrates significant differences in bacterial diversity, composition, and predicted functional potential between *T. mesenterina* and *C. thomasi* inhabiting a marginal, turbid, and bleaching-impacted reef system. Coral-associated microbiomes exhibited higher taxonomic richness, greater alpha diversity, and broader functional potential. In contrast, sponge microbiomes were less diverse but strongly dominated by a few taxa. Significant beta-diversity differences and distinct biomarker taxa revealed by LEfSe confirm that corals and sponges support distinct bacterial assemblages. Although both organisms experience similar environmental conditions, differences in host physiology, surface chemistry, and resource availability are likely key drivers of these differences in bacterial diversity. Understanding such host-specific microbiome signatures can inform predictions of benthic community shifts in marginal reefs and aid in the development of microbial indicators of reef health.

## Data Availability

The datasets presented in this study are publicly available. This data can be found at: https://www.ncbi.nlm.nih.gov/, accession number PRJNA917032.
