# Supplementary material for: Unraveling the bacterial composition of a coral and bioeroding sponge competing in a marginal coral environment
Source: Front Microbiol. 2025 Oct 2;16:1550446. doi: 10.3389/fmicb.2025.1550446 (PMC12529941; doi:10.3389/fmicb.2025.1550446)
Supplement: Supplementary file 1 [file Supplementary_file_1.docx]

**Unraveling bacterial composition of a coral and bio-eroding sponge competing in a marginal coral environment**

Sambhaji Mote^1,^, Kalyan De^1,2^, Mandar Nanajkar^1,2*^, Vishal Gupta^1,3*^

^1^CSIR–National Institute of Oceanography, Dona Paula, Goa-403004, India

^2^ Academy of Scientific and Innovative Research (AcSIR), Ghaziabad, 201002, India

^3^Climate Change Cluster, University of Technology Sydney, Australia

(Corresponding author: [mandar@nio.res.in](mailto:mandar@nio.res.in) & [vishal.gupta@uts.edu.au](mailto:vishal.k.gupta1983@gmail.com))

**Supplementary Table 1: Raw read summary of sequencing analysis**

| **Species** | **Sample ID** | **Raw reads** | **Filtered reads** | **Merged reads** | **Non-chimeric reads** |
| --- | --- | --- | --- | --- | --- |
| *Turbinaria mesenterina* | TM1 | 393349 | 227817 | 107914 | 97257 |
|  | TM2 | 335275 | 190386 | 82790 | 72870 |
|  | TM3 | 346252 | 199357 | 94346 | 85049 |
|  | TM4 | 308745 | 184108 | 61152 | 53748 |
|  | TM5 | 394587 | 230397 | 90018 | 79457 |
| *Cliona thomasi* | CT1 | 154833 | 91165 | 57934 | 46330 |
|  | CT2 | 166445 | 95737 | 58315 | 45867 |
|  | CT3 | 241813 | 141501 | 87585 | 77564 |
|  | CT4 | 212760 | 127765 | 83419 | 68293 |
|  | CT5 | 341386 | 201849 | 101929 | 91408 |

**Supplementary Table 2: Taxonomic assignment reads summary and alpha diversity indices**

| **Sample ID** | **Taxonomically assigned reads** | **Richness** | **Shannon** | **Simpson** |
| --- | --- | --- | --- | --- |
| **TM1** | 91186 | 1152 | 5.40 | 0.98 |
| **TM2** | 65235 | 1118 | 5.70 | 0.99 |
| **TM3** | 79192 | 1154 | 5.19 | 0.98 |
| **TM4** | 50832 | 1377 | 5.11 | 0.98 |
| **TM5** | 70093 | 1182 | 5.61 | 0.98 |
| **CT1** | 46090 | 260 | 3.61 | 0.94 |
| **CT2** | 45514 | 315 | 3.87 | 0.96 |
| **CT3** | 76632 | 402 | 3.59 | 0.93 |
| **CT4** | 61336 | 314 | 3.58 | 0.94 |
| **CT5** | 68419 | 339 | 3.51 | 0.93 |

**Supplementary Table 3: PARMANOA results indicates the differences among coral and sponge samples**

| Groups | df |  | *t*-value |  | *p*-value | Unique perms | P (MC) |
| --- | --- | --- | --- | --- | --- | --- | --- |
| *C. thomasi & T. mesenterina* | 8 |  | 2.4049 |  | 0.007 | 126 | 0.003 |
| Data were square-root transformed: *p*-value after permutation procedure and *p (MC)*-value based on Monte Carlo random draws | | | | | | | |

**Supplementary Table 4: The SIMPER analysis calculated the contribution of each species (%) to the dissimilarity between coral and sponge samples.**

| **Average dissimilarity: 81.44%** | | | | | |
| --- | --- | --- | --- | --- | --- |
| **Taxa** | ***C. thomasi*** | ***T. mesenterina*** | **Av.Dissim** | **Contrib%** | **Cum.%** |
| **Alphaproteobacteria** | 169.5 | 82.47 | 1.47 | 1.8 | 1.8 |
| **Synechococcus CC9902** | 61.74 | 27.04 | 0.54 | 0.66 | 2.47 |
| **Ruegeria** | 20.99 | 37.99 | 0.49 | 0.6 | 3.07 |
| **Blastopirellula** | 31.36 | 12.07 | 0.32 | 0.39 | 7.32 |
| **Leptospiraceae** | 37.65 | 10 | 0.45 | 0.56 | 4.21 |


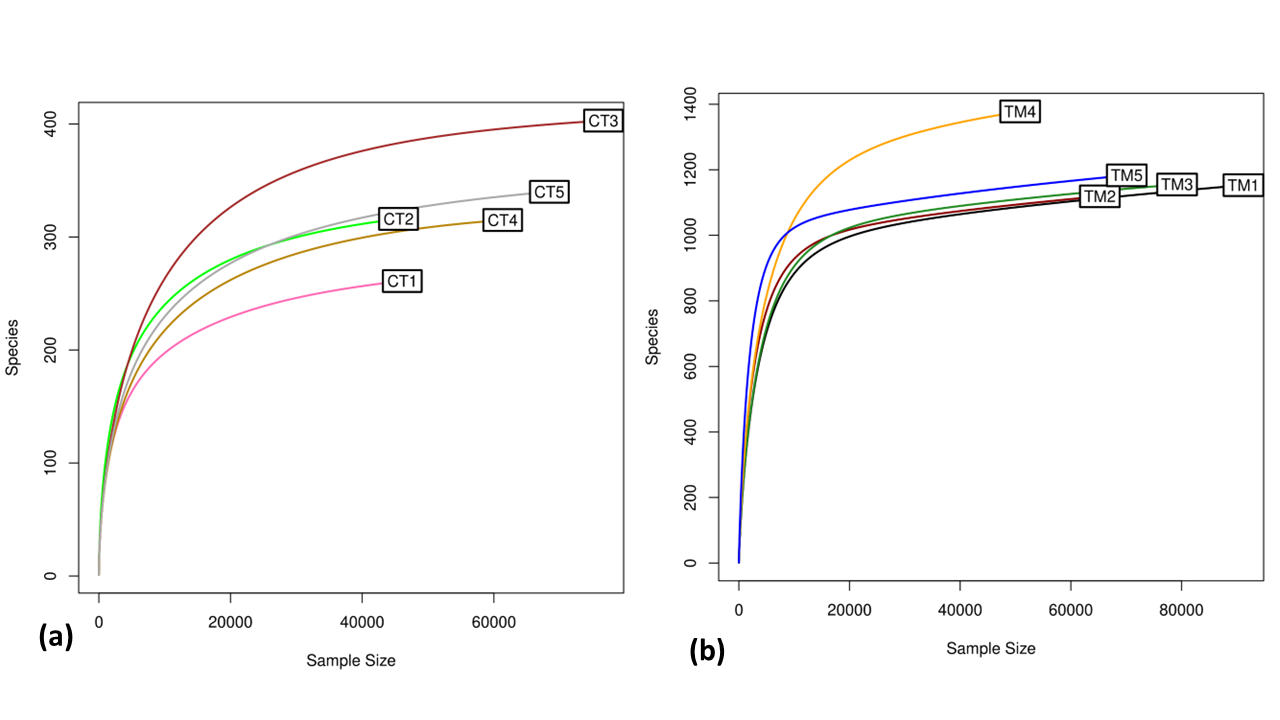


**Supplementary figure 1: Rare faction curve for coral showing higher abundance and richness in coral samples in comparison with the sponge samples (X axis: number of sequences read, Y axis: number of ASVs, a) Sponge samples, b) Coral samples.**
